# Understanding the current provisions of support for people with an intellectual disabilities and/or autism in crisis: A mixed methods study

**DOI:** 10.1177/00207640241303831

**Published:** 2024-12-09

**Authors:** Samuel Tromans, Ian Summers, Shahbaz Abdullah, Joanne Ledger, Sarah Lennard, Paul Bassett, Remie Colledge, Danielle Bilkey, Chloe Staples, Samuel Edwards, Grahame Carr, Richard Laugharne, Rohit Shankar

**Affiliations:** 1 SAPPHIRE Group, Department of Population Health Sciences, University of Leicester, UK; 2 Leicestershire Partnership NHS Trust, Leicester, UK; 3 Cornwall Intellectual Disability Equitable Research, Cornwall Partnership NHS Foundation Trust, Truro, UK; 4 Cornwall Intellectual Disability Equitable Research, University of Plymouth Peninsula School of Medicine, UK; 5 Statsconsultancy Ltd., Bucks, UK; 6 Expert by Experience (Independent), Coventry, UK; 7 NHS England South-West, Exeter, Devon, UK

**Keywords:** Intellectual disability, inpatient admission, crisis, emergency psychiatry, Blue-Light

## Abstract

**Background:**

There has been significant reduction in inpatient beds for people with intellectual disability and/or autism (PwID/A) in the UK in the last decade following high profile national scandals in specialist psychiatric hospitals. To reduce inappropriate admissions a new strategy (Blue-Light, an emergency multi-disciplinary meeting to prevent admission to hospital) was introduced. However, there is no research on the influence of Blue-Light on crisis management for PwID/A.

**Aim:**

To assess Blue-Light’s impact on PwID/A’s crisis presentations

**Methods:**

Co-produced with experts-by-experience, a mixed methods approach using a 13 question Likert based survey of health and social-care professionals along with semi-structured interviews of carers involved with consecutive Blue-Light patient reviews was undertaken in Cornwall UK (population: 538,000). Patient data was accessed to understand the patient journey. All data analysis was descriptive in nature. Semi-structured interviews were transcribed and thematically analysed using Braun and Clarke’s six-step process.

**Results:**

Ten patient journeys were examined. Staff interviewed had a good understanding of the Blue-Light process, Blue-Light activation practical challenges and considered Blue-Light reactive. Nearly half wanted ID/A specialist beds recommissioned. A majority wanted improved supervision and standards for third sector providers. Semi-structured interviews of 10 patient-carers identified a lack of consistency from professionals, limited infrastructure provision, the prolongation of crises and a lack of definition of crisis as carers did not feel supported by services.

**Conclusions:**

Current crisis support systems are not standardised and often leave carers feeling unsupported in crises. An evidence-based debate of crisis support and the inpatient role for PwID/A is required.

## Introduction

Over the past few decades, the United Kingdom (UK), particularly England, has undergone significant transformation to the way mental healthcare is provided to people with intellectual disability and/or autism (PwID/A; Burrows et al., 2023). Specialist hospital beds for PwID/A and co-occurring mental health needs and/or behavioural challenges have been systematically reduced (Burrows et al., 2023; Niven et al., 2020; Shankar, 2023). The public scrutiny of specialist inpatient psychiatric units that occurred after the Winterbourne View Scandal in 2011 was a catalyst for a major review of services for PwID/A leading to the Transforming Care report (Department of Health, 2012). The report focussed on enabling PwID/A who are admitted to inpatient psychiatric settings to move into the community, whilst also endeavouring to raise quality standards for inpatient admissions (Cooper et al., 2007; Department of Health, 2012). Following the launch of Transforming Care, the number of specialist mental health beds commissioned by Clinical Commissioning Groups reduced by 4.5% by the end of 2015, causing an almost 90% reduction in psychiatric beds for PwID/A in the NHS since 1987 (Devapriam et al., 2015; Public Health England, 2016). Whilst most PwID/A live independently, over a fifth (21%) still require contact with specialist psychiatric services sometime in their lifetime (Public Health England, 2016). A survey of consultant psychiatrists working with PwID/A in England showed 82% reported utilising specialist inpatient services on occasions for the better management of mental health and/or behavioural needs of some of their patients (Guinn et al., 2016).

There is a lack of ongitudinal data on the specific patient characteristics, and treatment outcomes, of those PwID/A accessing specialist inpatient settings, particularly post-Transforming Care (Painter et al., 2023). A recent study, the largest conducted to date, looking at 169 PwID/A admitted and discharged from a specialist unit between 2010 and 2018, showed treatment was effective across all causes of admission including those with co-occurring mental illness and behaviours that challenge (Quilca Espinoza et al., 2022). Treatment effectiveness was not associated with the duration of treatment suggesting events outside the locus of control of the psychiatric unit determined length of stay (Quilca Espinoza et al., 2022). These traces of evidence indicate that crisis admissions and then discharges for PwID/A into psychiatric units are a result of multi-factorial issues which have been inadequately researched (Shankar, 2023). Similar issues are prevalent in the community where evidence is lacking on what helps make successful community provision (Lennard et al., 2020; Shankar et al., 2015). Complex and vulnerable individuals are often discharged without adequate planning into regions away from their own place of origin impacting significantly on their wellbeing and ability of host services to cope (Shankar et al., 2015, 2016). Furthermore, there is evidence to suggest that locally available psychiatric units are presently ill-equipped to cope with the complex needs that PwID/A present with (Jones et al., 2021).

To mitigate these concerns, NHS England brought a policy termed Blue-Light (NHS England, 2015). Blue-Light has been developed as part of NHS England’s commitment to improving the care of PwID/A and with the aim of reducing admissions and unnecessarily lengthy stays in hospitals (NHS England, 2015). It requires stakeholders to prevent PwID/A being admitted unnecessarily into hospital beds by identifying barriers to supporting the PwID/A to remain in the community and to make clear and constructive recommendations to optimise their care and prevent future crises (NHS England, 2015). However, there is no research of Blue-Light’s influence on crisis management for PwID/A.

The aim of this study was to describe the experience of carers and staff in implementing the Blue-Light process for PwID/A in a crisis.

## Methodology

STROBE guidance for reporting cross sectional studies was used to design and report the study (Supplemental Information 1). Cornwall, a county based in south-west England, has a population of 538,000 and is one of the most socio-economically deprived areas in the UK and Northern Europe (Wikipedia, 2024). However, it was also one of the first places in the country to close all its specialist inpatient units for PwID/A in 2007 (Niven et al., 2020). Currently any admission for PwID/A uses local general psychiatric units and, rarely, out of county specialist hospital admissions in line with current best practice (NHS England, 2015; Walton et al., 2022).

### Co-production

The co-production was an equal shared partnership of people with lived experience navigators, carers and health and social care professionals working together, confirming the study protocol embeds the NIHR standards of best practice (National Institute for Health and Care Research, 2019). The Challenging Behaviour Foundation advised on the protocol. Cornwall Council Intellectual Disability and Autism Support team (CHAMPS) were in support to advise and guide accessible information.

### Ethics and governance

The Health Research Authority decision tool was tested to discover if NHS ethical approval was required. The decision tool indicated no ethical approval was required (Supplemental Information 2). The study was registered with the NHS Trust as a service evaluation and validated by the Trust Clinical Quality Improvement Group.

### Participants

Participants were all patient carers and their health and social care professionals involved with 10 consecutive patients who were referred for a Blue-Light review within the 1 year of the study commencement. Inclusion criteria for participants included being able to communicate with or without accessible information, being able to understand the participation information sheet, having received the survey (with consent implicit in their return) and that the person completing the semi-structured questions gives informed consent to participate in the interviews. Exclusion criteria included diagnosis with a significant co-occurring psychiatric condition that would limit the ability of the participant (carer) to take part in the study (e.g. psychosis and severe depression), and/or being unable to communicate with or without using accessible communication tools (e.g. British Sign Language, Makaton).

### Study design

The project methods and tools were co-produced with experts-by-experience. A mixed methods coordinated three stage approach (Martínez-Mesa et al., 2016) was used.

1. Patient data – in order to review the journey taken
2. Semi-structured interviews of carers involved with adult PwID/A in Blue-Light events to understand their experience
3. A 13 question Likert based survey of health and social care professionals who were linked to the patients focussed on in the Blue-Light meetings to understand their opinion.

#### Patient data

Patient data was accessed to understand the patient journey taken pre- and post Blue-Light alert. Reviewing of clinical care was effective in seeking and answering the aims of the service evaluation for people who cannot be interviewed due to clinical risk. The journey was guided by the structured Key Lines of Enquiry (KLOE, Supplemental Information 3) questions derived from NHSE (NHS England, 2023) with blended guidance embedded from the expert by experience. Key areas of focus included: the patient’s health check (and its date); pre-Blue-Light care, support and risk; when the crisis began; was support offered, accepted or declined pre- and post Blue-Light; was the person involved in the decision; what happened post Blue-Light; and was there a review post-crisis.

#### Professional carers interviews

Carers who supported a person through pre and post a Blue-Light alert were offered the opportunity to participate in a semi-structured interview. The semi-structured questionnaires were delivered to gain first-hand experiences pre and post the Blue-Light process by discovering service achievements and meaning for the carer. The carers were provided with the participant information sheet and supporting informed participation. A prerequisite to taking part in the study required the carers to give informed consent. The questionnaire (Supplemental Information 3) incorporated the KLOE semi-structured questions (Supplemental Information 3) by embedding the expectations of the Blue-Light meetings. It was developed with guidance from experts by experience. The interviewees were offered the opportunity of a face to face, video conference service or phone call interview, at a time that was suitable to them.

#### The health and social care professional’s survey

The group of social and health professionals who were involved in the Blue-Light patients care received a Likert scale survey of 13 questions (Supplemental Information 5). The time to complete the survey was 10 to 15 min. The data was collated anonymously, and recommendations supported an understanding of need and service development. The survey was co-produced with the aim of understanding the current service delivery and crisis timescale with the aim to discover if any barriers were present which could have affected outcomes. The email survey was sent by NHS secure email to all key professionals who had direct or indirect involvement in the identified patients Blue-Light in Cornwall. The participant information sheet discusses explicit consent and informed participants that their survey data would be anonymous. Consent was presumed by reply to survey.

### Data analysis

A cconvergent parallel mixed-methods approach was employed, as neither a qualitative nor a quantitative approach alone would be sufficient to answer the study’s aims. The proactive triangulation of the systemic, procedural and contextual data has informed the design and implementation of effective interventions. The investigative phase captured codes which were framed and the analytical significance was discussed and agreed by three researchers in parallel with the intercoder reliability fluid flow chart (O’Connor & Joffe, 2020). All data analysis was descriptive in nature. Categorical variables were summarised by the number and percentage of responses in each category, whilst continuous variables were summarised by the mean and standard deviation.

Semi-structured interviews were completed remotely, and differences reconciled before conducting a thematic analysis following Braun and Clarke’s six-step process (Braun & Clarke, 2006). The deductive and inductive were employed together to enhance the understanding of the topic to generate themes, which were transcribed by three research analysts, so reflecting on both prior knowledge by the researchers and the development of themes which naturally emerged from the data.

## Results

### Demographic data

**Figure 1.**
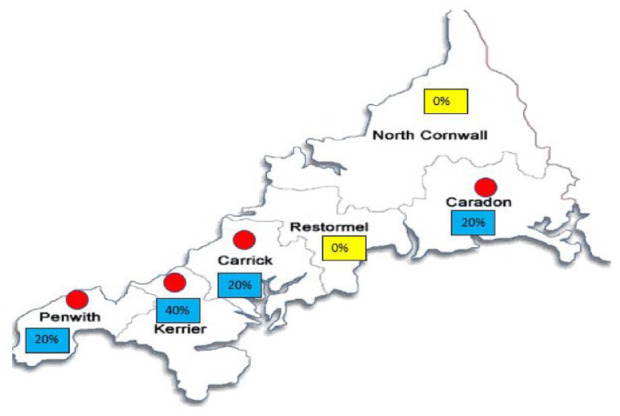
Distribution of participants.

The postcode demographic data map (Figure 1) highlights the variation of Blue-Light alerts across the six districts of Cornwall, with some regions having no alerts raised. There may be an association of Blue-Light alerts to the most deprived areas of Cornwall (Supplemental Information 6).

### Patient data

The characteristics of the patients are summarised in Table 1. From the study population, 8 were male, of whom 7 were within the 18 to 30 years age group, 7 were unemployed and all 10 were single. Of the 10, the primary need for 6 was their ID and for 4 autism spectrum disorder. With respect to co-occurring major psychiatric diagnoses, six had anxiety, five had depression and two had schizophrenia. For all 10 patients, increased challenging behaviour, mental health difficulties and risk were the precipitating factors for crisis (Table 2).

**Table 1.** Summary of patient participant characteristics.

Factor	*n*	Category	Number (%)
Gender	10	Female	1 (10)
		Male	8 (80)
		Non-gender	1 (10)
Age group (years)	10	18–30	7 (70)
		31–40	1 (10)
		41–50	2 (20)
Type of care	10	Family	6 (60)
		Private care	4 (40)
Accommodation	10	Mum/Dads home	6 (60)
		Supportive	4 (40)
Marital status	10	Single	10 (100)
		Married	0 (0)
Employment	10	Part time employment	1 (10)
		Volunteering	1 (10)
		Student	1 (10)
		Seeking work	2 (20)
		Unemployed	5 (50)
Psychiatric diagnoses	10	Anxiety	6 (60)
		Depression	5 (50)
		Schizophrenia	2 (20)
		Borderline personality disorder	3 (30)
		Bipolar affective disorder	0 (0)
		Other diagnosis	5 (50)
ID	10	ID present	6 (60)
		Mild ID	2 (20)
		Moderate/Profound ID	4 (40)
Autism	10	Autism present	10 (100)
		Level 1	0 (0)
		Level 2	6 (60)
		Level 3	4 (40)
		Both autism and ID present	6 (60)
Factors contributing to crisis	10	Support breakdown	5 (50)
		Challenging behaviour	10 (100)
		Accommodation	8 (80)
		Risk increases	10 (100)
		Mental health difficulties	10 (100)

**Table 2.** Staff survey respondents and response summary.

Variable	*n*	Category	Number (%)
Staff profession	17	Administration	1 (6%)
		Mental health liaison	1 (6%)
		Nurse	4 (24%)
		Occupational therapist	2 (12%)
		Outreach worker	1 (6%)
		Physiotherapist	1 (6%)
		Psychiatrist	1 (6%)
		Psychologist	3 (18%)
		Social worker	1 (6%)
		Speech and language therapist	1 (6%)
		Therapist/RMN	1 (6%)
Blue-light process			
Understand role within Blue-Light process	17	Strongly disagree	1 (6%)
		Disagree	1 (6%)
		Neither agree/disagree	2 (12%)
		Agree	8 (47%)
		Strongly agree	5 (29%)
Can respond to Blue-Light event	17	Strongly disagree	0 (0%)
		Disagree	2 (12%)
		Neither agree/disagree	4 (24%)
		Agree	6 (35%)
		Strongly agree	5 (29%)
Unable to support Blue -Light process	17	Strongly disagree	6 (35%)
		Disagree	4 (24%)
		Neither agree/disagree	1 (6%)
		Agree	4 (24%)
		Strongly agree	2 (12%)
Crisis identified earlier	17	6 months prior	1 (6%)
		3 months prior	1 (6%)
		1 month prior	9 (53%)
		1 week prior	4 (24%)
		No signs notified	1 (6%)
		No involvement	1 (6%)
Disability training and QNLD standards			
ID training	15	No	5 (33%)
		Yes	10 (67%)
Aware of QLND standards	17	Strongly disagree	2 (12%)
		Disagree	3 (18%)
		Neither agree/disagree	0 (0%)
		Agree	9 (53%)
		Strongly agree	3 (18%)
Able to embed QLND standards into role	17	Strongly disagree	2 (12%)
		Disagree	2 (12%)
		Neither agree/disagree	3 (18%)
		Agree	7 (41%)
		Strongly agree	3 (18%)
Green light agenda and mental health inpatient services			
Good awareness of green light agenda	17	Strongly disagree	2 (12%)
		Disagree	1 (6%)
		Neither agree/disagree	1 (6%)
		Agree	8 (47%)
		Strongly agree	5 (29%)
Green light agenda lost focus on improvement	17	Strongly disagree	0 (0%)
		Disagree	3 (12%)
		Neither agree/disagree	9 (53%)
		Agree	2 (35%)
		Strongly agree	0 (0%)
Nurses should be flexible in service transfer	17	Strongly disagree	1 (6%)
		Disagree	3 (18%)
		Neither agree/disagree	2 (12%)
		Agree	6 (35%)
		Strongly agree	5 (29%)
Social care and IST team			
Believe ID beds be recommissioned	17	Strongly disagree	2 (12%)
		Disagree	5 (29%)
		Neither agree/disagree	2 (12%)
		Agree	3 (18%)
		Strongly agree	5 (29%)
LD nurses embedded in MH services as standard	17	Strongly disagree	0 (0%)
		Disagree	2 (12%)
		Neither agree/disagree	0 (0%)
		Agree	7 (41%)
		Strongly agree	8 (47%)
Reactive model fails to react to change	17	Strongly disagree	0 (0%)
		Disagree	1 (6%)
		Neither agree / disagree	2 (12%)
		Agree	9 (53%)
		Strongly agree	5 (29%)
Should reactive model revert	17	Strongly disagree	0 (0%)
		Disagree	1 (6%)
		Neither agree/disagree	3 (18%)
		Agree	6 (35%)
		Strongly agree	7 (41%)
Should use a joint proactive model	17	Strongly disagree	0 (0%)
		Disagree	1 (6%)
		Neither agree/disagree	1 (6%)
		Agree	3 (18%)
		Strongly agree	12 (71%)
IST reduced ability has increased risk of escalation	17	Strongly disagree	1 (6%)
		Disagree	1 (6%)
		Neither agree/disagree	6 (35%)
		Agree	5 (29%)
		Strongly agree	4 (24%)
IST move to 24/7 service	17	Strongly disagree	1 (6%)
		Disagree	4 (24%)
		Neither agree/disagree	3 (18%)
		Agree	4 (24%)
		Strongly agree	5 (29%)
IST response ability based in 24/7 support hub	17	Strongly disagree	1 (6%)
		Disagree	7 (41%)
		Neither agree/disagree	4 (24%)
		Agree	4 (24%)
		Strongly agree	1 (6%)
Community provision and rating of working relationships			
Suitability of community provision increases risk	17	Strongly disagree	0 (0%)
		Disagree	0 (0%)
		Neither agree/disagree	2 (12%)
		Agree	5 (29%)
		Strongly agree	10 (59%)
Community providers should increase notice period	17	Strongly disagree	0 (0%)
		Disagree	0 (0%)
		Neither agree/disagree	3 (18%)
		Agree	3 (18%)
		Strongly agree	11 (65%)
Limited oversight increases risk of safe care	17	Strongly disagree	0 (0%)
		Disagree	1 (6%)
		Neither agree/disagree	1 (6%)
		Agree	5 (29%)
		Strongly agree	10 (59%)
Rating of relationship between health and social care	17	–	5.7 ± 2.4

**Figure 2.**
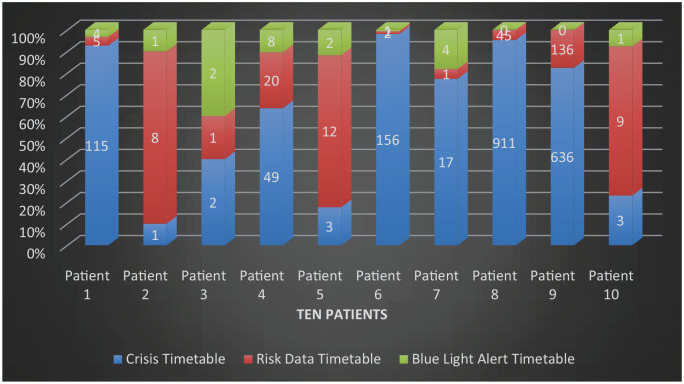
Crisis timetable graph (in days). *Note*. Blue + red + green = covers from start of crisis for the individual to the time of blue-light meeting. Blue – the duration in % of time the person was in crisis till a health or social care professional was notified. Red – the duration in % of time the person was in crisis till the health and social care staff took action of putting a blue-light referral. Green – the duration in % of time the referral took to get processed for the individual.

Figure 2 expresses the duration people remained in crisis up to a Blue-Light meeting occurred. People’s risk assessments when in ‘red’ were not always updated with the increasing risk. However, once the Blue-Light referral in Green was completed, the Blue-Light emergency meeting took place on average within 36 hours of receiving a referral.

### Staff survey

Responses were received from a total of 17 staff, including 4 nurses, 3 psychologists and 1 social worker. The numbers of each type are summarised in Table 1, along with staff survey responses.

Three quarters of respondents felt they had a good awareness of the ‘Green Light’ agenda, that is, around reasonable adjustments to accommodate PwID/A in general psychiatric inpatient units. There was no strong consensus as to whether the agenda had lost focus on improvement, with nine staff members giving a neutral opinion and no staff either strongly agreeing or strongly disagreeing. Nearly two-thirds of respondents (11, 64%) agreed that staff should be flexible by transferring from community to inpatient wards.

There was a divided opinion on whether specialist beds for PwID/A be recommissioned in Cornwall, with eight (47%) staff in favor and seven (41%) not. There was more agreement on specialist nurses in ID being embedded in mental health services, with 15 (88%) respondents agreeing that this was a good idea. A majority of staff, that is, 14 (82%) felt that the current crisis support model fails to react to change with three-quarters (13, 76%) feeling that the reactive model should revert to a more proactive prevention model. Additionally, 15 (88%) staff supported a joint proactive model should be used.

Most respondents (15, 88%) believed private provider led community provision suitability negatively influenced the crisis risk. Most staff (14, 82%) also thought that community providers should increase the notice period given when a patient goes into crisis. The view that limited professional oversight increases the risk of care was felt by 15 (88%) staff. When asked to give a rating of the strength of the relationship between health and social care in the local community on a scale of 0 to 10 (0 being very bad to 10 being good), the mean value was 5.7 (standard deviation 2.4). Range of individual staff rating was from 0 to 10. A summary of recommendations emerging from the clinicians and social care professionals is provided in Supplemental Information 7.

### Semi-structured interview of patient carers

From the semi-structured interviews conducted with 10 carers, four themes were identified: lack of consistency, limited provision of infrastructure, prolonging of crisis and definition and understanding of crisis.

#### Theme 1: Lack of consistency

Carers reported inconsistency and varied experiences in understanding the Blue-Light process and how this will impact on the person they supported. Whilst there were some positive experiences when a Blue-Light was called, there was limited prior understanding or training of what a Blue-Light is and how this will affect the carer or person before a crisis emerged and post-crisis.

*We didn’t know what had happened. All that we were told was that they were going to call a Blue-Light meeting because of X’s behaviour.*

It was reported information from the Blue-Light meeting was not always shared with carers and they felt they did not know the outcome or what would change in support or be reviewed post Blue-Light.

*We never got minutes from the meeting. So, I don’t really know. Nothing. Nothing.*

Whilst carers were encouraged to attend the Blue-Light meetings, they reported they received no support in attending a face-to-face meeting and felt that their other caring duties and employment responsibilities were not always considered. Furthermore, carers felt that the Blue-Light meetings did not always encourage a face-to-face discussion, with a remote meeting being the main preference by health and social care professionals when arranging this at short notice.

*They kept putting the meetings at times when they knew I couldn’t attend and then they’d say can you attend over the phone. I had my carer duties and no support to look after X while I attend.*

However, one person indicated that they did receive support in attending, whereby the Blue-Light was organised around their employment hours.

The advocacy support was not always available to the patient and carer during a crisis; however, the importance of advocacy support became evident in how people and carers received support during the Blue-Light crisis. One carer emphasised the importance of advocacy and without the advocate’s support they would not be able to manage the crisis.

*To be fair, I was in such a state. I was just all over the place at the time. The advocate took over when I was unable to take part. Oh my goodness. She was such a support. She was amazing. I couldn’t. I wouldn’t be here without her help.*

#### Theme 2: Limited provision of infrastructure

The service evaluation highlighted the range of additional and specialist support a PwID/A may need to stay out of hospital. However, too frequently this additional support is not available and further compacted with a short period notice of the private provider of one month to find alternative support/accommodation when transferring from one private service to another. An example is of one patient receiving emergency accommodation in a public house bed and breakfast due to their accommodation breakdown. However, this arrangement broke down soon, leading to the person being moved to temporary holiday accommodation ending up in further difficulties.

There is varied experience when seeking support and this has caused person and carer frustrations. However, some carers have voiced their support for the Blue Light chair/team and their honesty and openness. It was felt they tried their best with the limited provisions available to them.

*Blue-Light Meeting Chair did everything that they said they were going to do. So yeah, I can’t fault them. They tried their best.*

However, some reported they were left with limited support or no support and not all agreed/appropriate support could be or was delivered.

#### Theme 3: Prolonging of crisis

Carers expressed frustration in coping alone during the crisis, since carers were advised to contact several crisis lines for support by health and social care professionals, but at times were unable to connect or some contact numbers did not work or the call led to no significant offer of support.

*We really did need the help urgently because, like, he was breaking everything in sight. Two weeks was too long. But I do understand they had to sort of get plans and speak to people to put things together for him.*

At times the advice was to call the police for advice and support.

*They basically just told me if it carried on, things escalated to call the police or hung up after 20 minutes with no support.*

Carers were concerned the crisis support needed was not available and wondered why the crisis team did not respond. This led to over-reliance on the police to provide the advice and support needed during their crisis. The police became the carers main support before a Blue-Light referral was made. The carers have highlighted the positive support received from police during the crisis, who attempted to get support for patients

*They were very supportive. They tried pushing for help so many times. I’ve seen them about 40 times now. I’m on first name terms with some of them. That’s how bad it’s got.*

#### Theme 4: Definition and understanding of a crisis

Crisis lacked definition and there was lack of clarity on who provides crisis service provision. Carers reported they were at breaking point and emotionally distressed as the risk heightened with some support being offered that did not materialise.

*We were promised sort of intense support team input for X maybe once or twice a week, never happened.*

At times carers repeatedly requested help in supporting the person, however the crisis was not always acknowledged, and the Blue-Light referrals were at times delayed.

*I just think it is reactive. It’s too late, since we are in crisis and still, we wait for help.*

A carer highlighted communication issues of patients not being listened to.

*It was clear the pain X was experiencing was the reason why X was trying to get help but was unable to express it through his communication difficulties, but no one picked it up.*

The person received an anti-social behaviour order (ASBO), requiring the person to only visit the local hospital for appointments or emergency care. The carer felt no one was listening or understanding the crisis and felt abandoned with no support or information available in helping the person understand the ASBO.

*Suddenly we got a knock on the door and there was the community police officer with the anti-social person.*

However, the person continued to experience distress and called an ambulance, which the carer became frightened of the consequences the ASBO would have on the person and tried to prevent the person requesting an ambulance to go to hospital. The person attended the hospital, and it became clear they needed an emergency operation, since the behaviours that challenged the person were identified as an expression of pain.

*This sudden emergency operation, then he was really ill again afterwards, and we were terrified when he was calling an ambulance because he felt so unwell. And my husband and I are going. X, you can’t. You might get into trouble.*

The carer reported once it was discovered the behaviours observed were the person expressing pain, the ID/autism team stepped in and made the experience in hospital a positive experience. However, it took a lot of effort and multiple crises to get to this satisfactory phase.

*What’s happened since he’s been in hospital is that I’ve spoken to the disabilities team and autism team at the hospital have been brilliant.*

I think, I mean I don’t know that they do this is you’ve hit crisis, but you shouldn’t even have to get to that awful crisis point.

## Discussion

This study endeavoured to conduct a detailed assessment of participants journeys when referred for Blue-Light meetings using reviews of clinical records, record, surveys of staff and in-depth interviews of carers. It sought to identify the impact (or lack of impact) of the Blue-Light process on professionals, carers and patients.

The lack of standardisation in the Blue-Light process to provide or define resources in a stretched health and social care environment is evident. Further, Blue-Light processes appear to be defined by a reactive model of service delivery with no obvious pathways or references to provide a proactive response during crisis, which can impede care. The reviewed data has further indicated the difficulties health and social care staff continue to experience in accessing the right support for short- and long-term care. Health and social care professionals have voiced the need to recommission ID/A specialist beds with improved community provision. Community provision needs to be quality tested, indicating the quality-of-service provision, since there is a belief there is limited oversight of community provision impacting on the quality of service provided in crisis.

Health and social care professionals have expressed a need to increase notice periods of private providers from the current one month to allow for the right support to be found especially given the current limited resources. The National Autistic Society has highlighted the same concerns in a recent report and stated there is an urgent need for further Government funding to deliver effective community health and social care services (National Autistic Society, 2023). Without it, PwID/A are at risk of being in a vicious circle of ongoing crisis.

### Lack of consistency from professionals

There was recognised confusion and ambiguity to identify when a Blue-Light referral threshold had been met, especially for professionals. This highlights a need for an objective, valid and reliable measure to determine when this threshold has been met, to reliably identify patients in need of a Blue-Light referral.

### Limited infrastructure provision

Capacity to attend a Blue-Light needs to be considered, since some professionals reported they did not always have capacity to attend or had to cancel other important activities. Carers and professionals both struggled at times to access information about Blue-Light and when the process was activated, and there was no impetus to provide person-centred information pre- and post-meeting. Information regarding the Blue-Light and who to contact when a crisis happens needs a process where this information is readily available pre- and post Blue-Light.*The prolongation of crises*

The carers’ experience and reviewed data highlighted people faced prolonged periods of crisis and limited access to community support. Health and social care professionals indicated that people in their care experienced a varied period of crisis lasting between 1 and 24 weeks before a Blue-Light referral was made.

### Lack of definition of crisis

There were many highlighted concerns of how crisis was defined, which were voiced by carers, health and social care professionals and was also highlighted within the reviewed patient data. The risks had not always been defined, limiting understanding of what the threshold of crisis is and who provides support. This means that Blue-Light referrals were likely to be unstructured and ambiguous at times. The review of the crisis and support given needs to be completed in collaboration with the carer, to understand if the support is working and is suitable to the needs of the person and carer.

### Perspective of our expert with experience of the study findings

The findings of the study align with certain aspects of my own lived experience. They provide valuable insights that I hope will spark changes to the Blue Light protocol, and a shift towards a more proactive, transparent and accessible approach to reviewing care and support needs of autistic people and PwID. Blue Light meetings often happen at short notice to help prevent an admission to hospital. The question that I keep coming back to in my mind is, does this give the individual and their family or carers, the best possible chance to be involved in this process and to work together with members of care and support staff, or does its risk excluding them from crucial conversations? From experience, the further a crisis escalates, the more difficult working together and building trusting relationships becomes. When meetings are scheduled at short notice, this often does not provide sufficient time to prepare emotionally or practically, adding to stress and overwhelm levels. It may not allow enough time for reasonable adjustments to be made to support people to attend, reinforcing some of the inequalities reflected in wider society. If review meetings can take place at an earlier point, I feel it would maximise the opportunity to work together to problem solve and think of creative and innovative ideas to best support the individual at the heart of the decision-making process. Reflecting on the findings, my second question is, how can information about Care and Treatment reviews and Blue Light meetings be made more accessible, supportive and transparent for both the individual, but also their family or carers? The findings reflect that there can be a lack of information about Blue Light processes. Often, I found the language and information in and around being on the Dynamic Support Register (DSR) very clinical and confusing, and it caused me a significant degree of anxiety. It was only after life moved forward and I no longer needed to be on the DSR, that this anxiety lifted and that I fully understood the value it had had within my own recovery. Clear, jargon-free language, people taking the time to explain what is happening or at least explaining why something could not happen at the earliest possible opportunity, and receiving summarised minutes and outcomes, can be powerful ways of providing consistency and making sure everyone understands what is happening. Finally, I vividly recall one of the most transformative meetings I had within my recovery, a Care and Treatment Review. There was an Expert by Experience present, I received information beforehand and was sent the outcomes in writing afterwards. I use the word ‘transformative,’ not because of the outcomes, but because of the way the meeting was planned and facilitated, allowing me to feel heard, included and valued’.

### Limitations

Patients were not interviewed directly. This is because there were concerns of risks of traumatising the person, since many were still likely to be still in crisis during the service evaluation. Thus, surrogate information from carers was collected. Not having the voice of the person within the service evaluation is acknowledged as a limitation. However, the results of the service evaluation is expected to offer patients future research opportunities and a potential national study to discover if the results found in the service evaluation need further investigation.

Response rates from clinical employees achieved a 53% return; however, the response rate from social care-related workers was 25%. Understanding the perspective of social care employees is limited in this sample. The carers semi-structured interview achieved a 20% participation. However, the convergent parallel data from surveys, interviews and reviewed patient records identified a point of saturation, thus an adequate sample size was achieved to evaluate the combined data (Hennink & Kaiser, 2022).

Finally, the sample size is relatively small, and as such quantitative data was presented descriptively rather than subject to significance testing. Additionally, there is a lack of data relating to patient ethnicity, and all patients were localised to Cornwall; thus we cannot be confident that the findings reported here are generalisable on a national level.

## Conclusion

This service evaluation could be replicated in different services to develop an understanding from those who use, depend on and those who work in service delivery, to explore how these findings vary across other parts of the country particularly in the Blue-Light process.

Our findings suggest that the Blue-Light process lacks a standardised model resulting in a service which is ill-defined. Carers often feel excluded from the process and abandoned by services when PwID/A are in crisis. Often they have to rely on the police for support.

The implications for research is the need to thoroughly and dispassionately define models of care for PwID/A in crisis and then evaluate them to build an evidence base. The implications for clinical care are the need for clinicians to describe models of care that they have developed so that these can be evaluated and implemented for patients. For policy, health departments should be seeking models of crisis care that are evaluated and encourage their implementation, and then use service evaluations to establish if the care improves. Policy should be driven by evidence and not ideology. Closing inpatient beds may have been driven too much by ideology without a close examination of how to care for PwID/A in crisis. We owe it to patients who are too often neglected when in crisis not to make the same mistake again.

## Supplemental Material

sj-docx-1-isp-10.1177_00207640241303831 – Supplemental material for Understanding the current provisions of support for people with an intellectual disabilities and/or autism in crisis: A mixed methods study Supplemental material, sj-docx-1-isp-10.1177_00207640241303831 for Understanding the current provisions of support for people with an intellectual disabilities and/or autism in crisis: A mixed methods study by Samuel Tromans, Ian Summers, Shahbaz Abdullah, Joanne Ledger, Sarah Lennard, Paul Bassett, Remie Colledge, Danielle Bilkey, Chloe Staples, Samuel Edwards, Grahame Carr, Richard Laugharne and Rohit Shankar in International Journal of Social Psychiatry

sj-docx-2-isp-10.1177_00207640241303831 – Supplemental material for Understanding the current provisions of support for people with an intellectual disabilities and/or autism in crisis: A mixed methods study Supplemental material, sj-docx-2-isp-10.1177_00207640241303831 for Understanding the current provisions of support for people with an intellectual disabilities and/or autism in crisis: A mixed methods study by Samuel Tromans, Ian Summers, Shahbaz Abdullah, Joanne Ledger, Sarah Lennard, Paul Bassett, Remie Colledge, Danielle Bilkey, Chloe Staples, Samuel Edwards, Grahame Carr, Richard Laugharne and Rohit Shankar in International Journal of Social Psychiatry

sj-docx-3-isp-10.1177_00207640241303831 – Supplemental material for Understanding the current provisions of support for people with an intellectual disabilities and/or autism in crisis: A mixed methods study Supplemental material, sj-docx-3-isp-10.1177_00207640241303831 for Understanding the current provisions of support for people with an intellectual disabilities and/or autism in crisis: A mixed methods study by Samuel Tromans, Ian Summers, Shahbaz Abdullah, Joanne Ledger, Sarah Lennard, Paul Bassett, Remie Colledge, Danielle Bilkey, Chloe Staples, Samuel Edwards, Grahame Carr, Richard Laugharne and Rohit Shankar in International Journal of Social Psychiatry

sj-docx-4-isp-10.1177_00207640241303831 – Supplemental material for Understanding the current provisions of support for people with an intellectual disabilities and/or autism in crisis: A mixed methods study Supplemental material, sj-docx-4-isp-10.1177_00207640241303831 for Understanding the current provisions of support for people with an intellectual disabilities and/or autism in crisis: A mixed methods study by Samuel Tromans, Ian Summers, Shahbaz Abdullah, Joanne Ledger, Sarah Lennard, Paul Bassett, Remie Colledge, Danielle Bilkey, Chloe Staples, Samuel Edwards, Grahame Carr, Richard Laugharne and Rohit Shankar in International Journal of Social Psychiatry

sj-docx-5-isp-10.1177_00207640241303831 – Supplemental material for Understanding the current provisions of support for people with an intellectual disabilities and/or autism in crisis: A mixed methods study Supplemental material, sj-docx-5-isp-10.1177_00207640241303831 for Understanding the current provisions of support for people with an intellectual disabilities and/or autism in crisis: A mixed methods study by Samuel Tromans, Ian Summers, Shahbaz Abdullah, Joanne Ledger, Sarah Lennard, Paul Bassett, Remie Colledge, Danielle Bilkey, Chloe Staples, Samuel Edwards, Grahame Carr, Richard Laugharne and Rohit Shankar in International Journal of Social Psychiatry

sj-docx-6-isp-10.1177_00207640241303831 – Supplemental material for Understanding the current provisions of support for people with an intellectual disabilities and/or autism in crisis: A mixed methods study Supplemental material, sj-docx-6-isp-10.1177_00207640241303831 for Understanding the current provisions of support for people with an intellectual disabilities and/or autism in crisis: A mixed methods study by Samuel Tromans, Ian Summers, Shahbaz Abdullah, Joanne Ledger, Sarah Lennard, Paul Bassett, Remie Colledge, Danielle Bilkey, Chloe Staples, Samuel Edwards, Grahame Carr, Richard Laugharne and Rohit Shankar in International Journal of Social Psychiatry

sj-docx-7-isp-10.1177_00207640241303831 – Supplemental material for Understanding the current provisions of support for people with an intellectual disabilities and/or autism in crisis: A mixed methods study Supplemental material, sj-docx-7-isp-10.1177_00207640241303831 for Understanding the current provisions of support for people with an intellectual disabilities and/or autism in crisis: A mixed methods study by Samuel Tromans, Ian Summers, Shahbaz Abdullah, Joanne Ledger, Sarah Lennard, Paul Bassett, Remie Colledge, Danielle Bilkey, Chloe Staples, Samuel Edwards, Grahame Carr, Richard Laugharne and Rohit Shankar in International Journal of Social Psychiatry
