# Supplementary figures and images for: Understanding the current provisions of support for people with an intellectual disabilities and/or autism in crisis: A mixed methods study
Source: Int J Soc Psychiatry. 2024 Dec 9;71(4):782–93. doi: 10.1177/00207640241303831 (PMC12171051; doi:10.1177/00207640241303831)

## **Supplementary Information 2: Ethics Decision Tool**

**
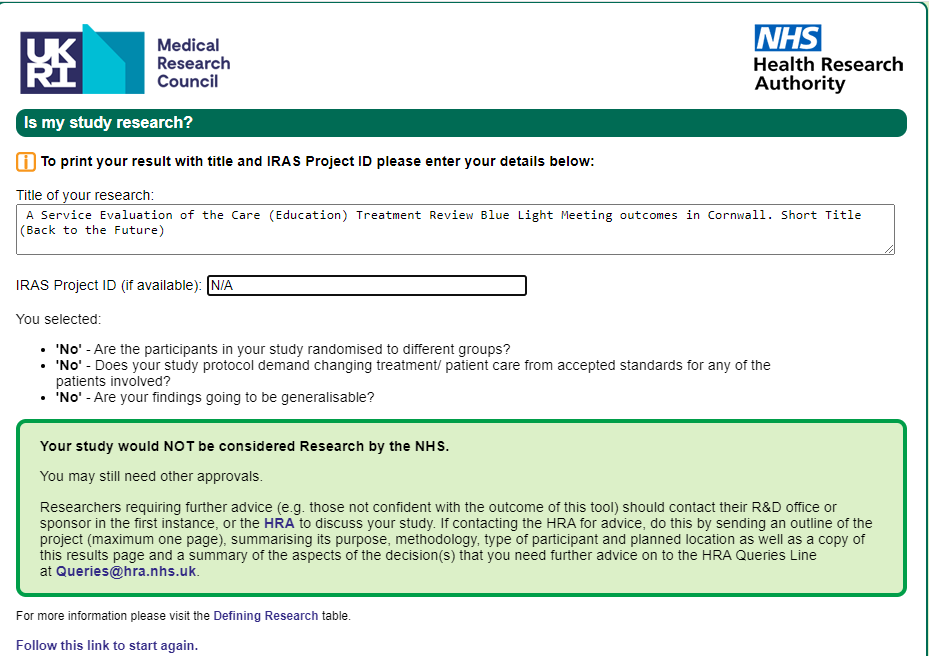
**

Supplement: sj-docx-2-isp-10.1177_00207640241303831 – Supplemental material for Understanding the current provisions of support for people with an intellectual disabilities and/or autism in crisis: A mixed methods study [file sj-docx-2-isp-10.1177_00207640241303831.docx]

**Supplementary Information 4: Carers Survey**

**
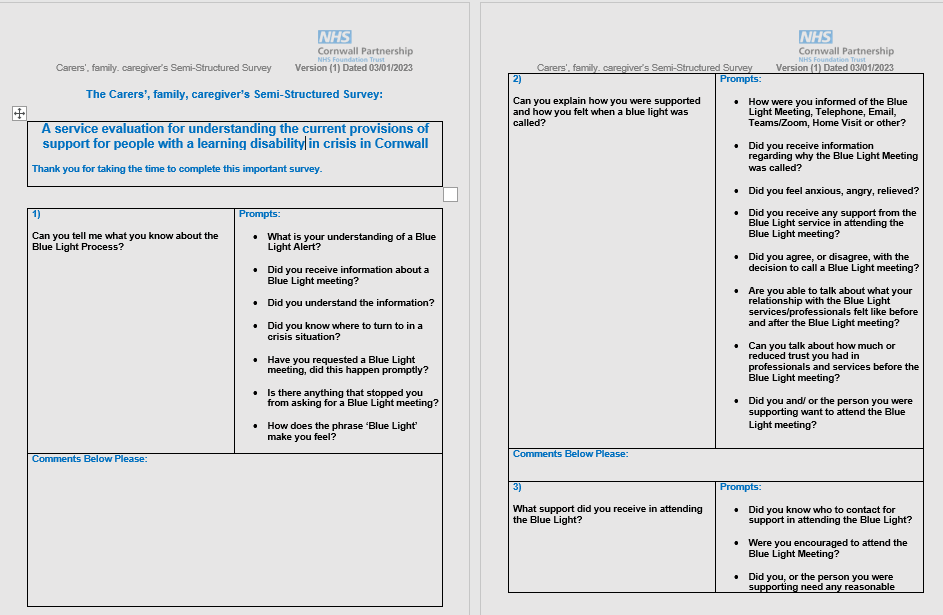
**

**
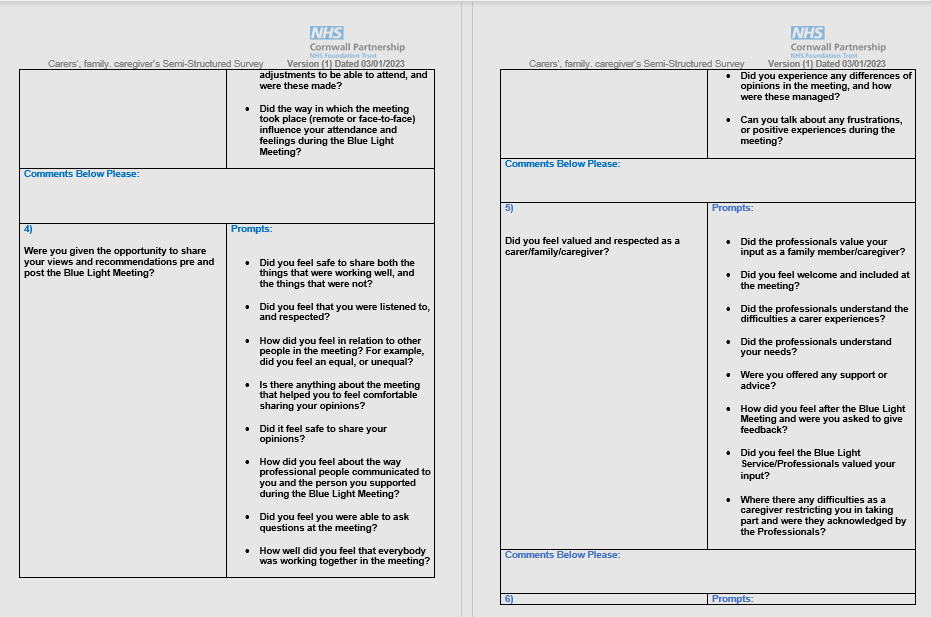
**

**
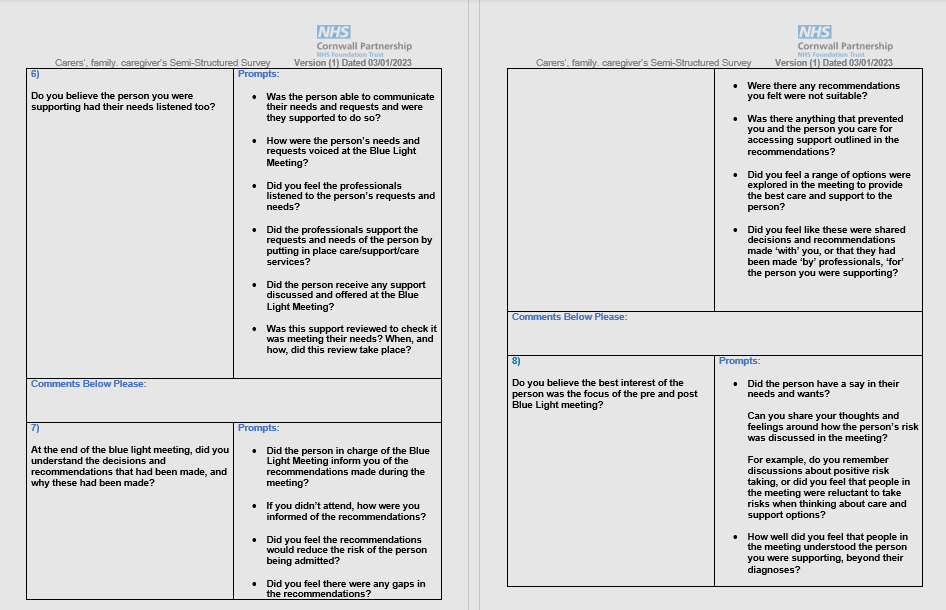
**

**
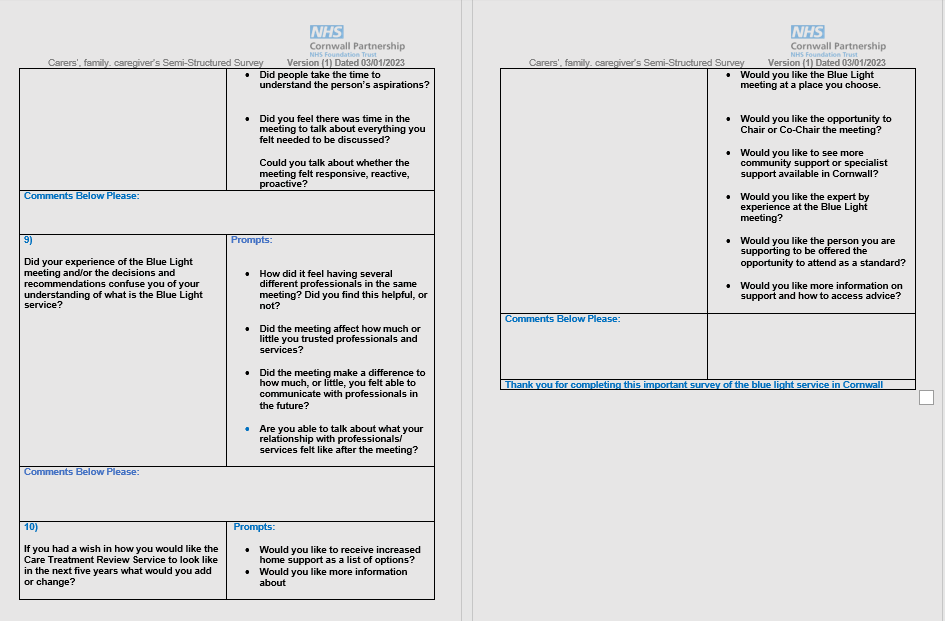
**

Supplement: sj-docx-4-isp-10.1177_00207640241303831 – Supplemental material for Understanding the current provisions of support for people with an intellectual disabilities and/or autism in crisis: A mixed methods study [file sj-docx-4-isp-10.1177_00207640241303831.docx]

**Supplementary information 5: Health Professionals Survey**

**
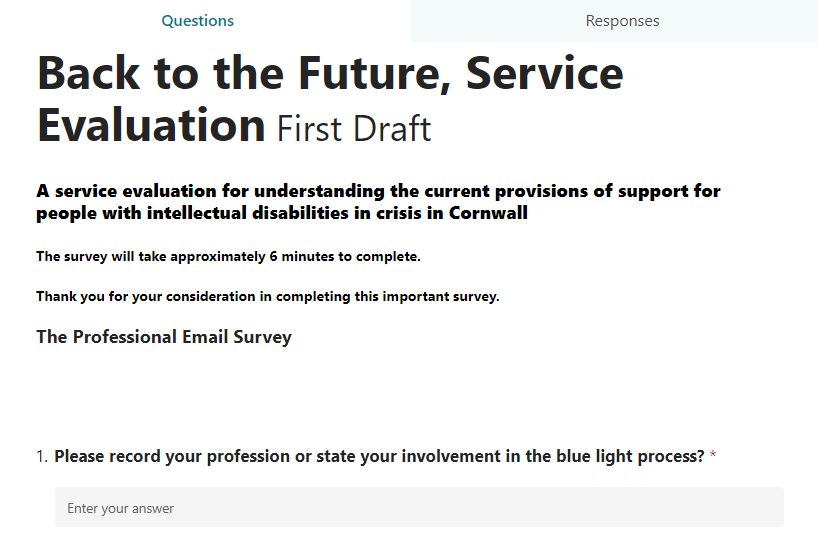
**

**
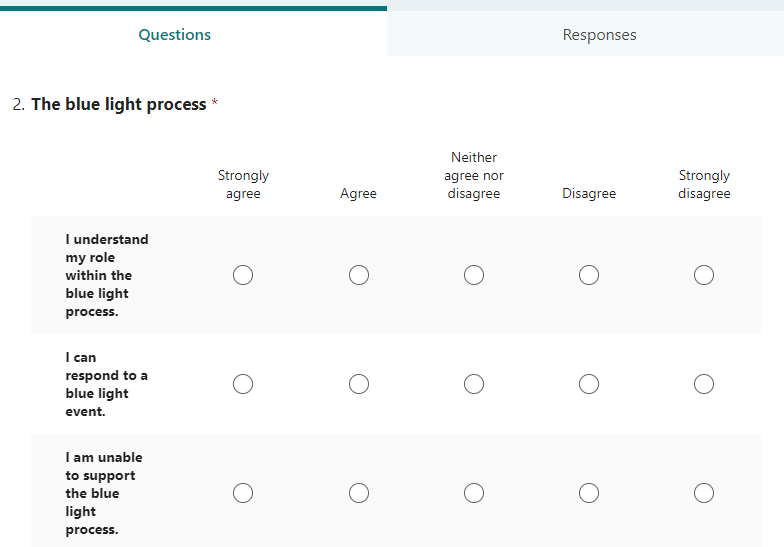
**

**
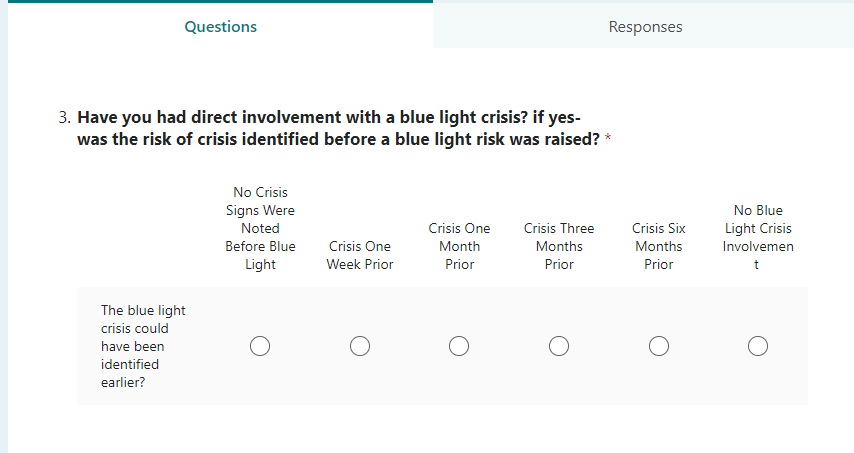
**

**
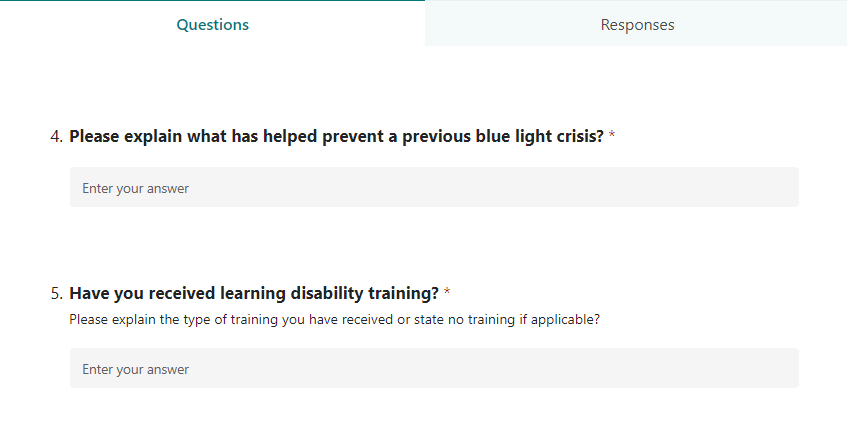
**

**
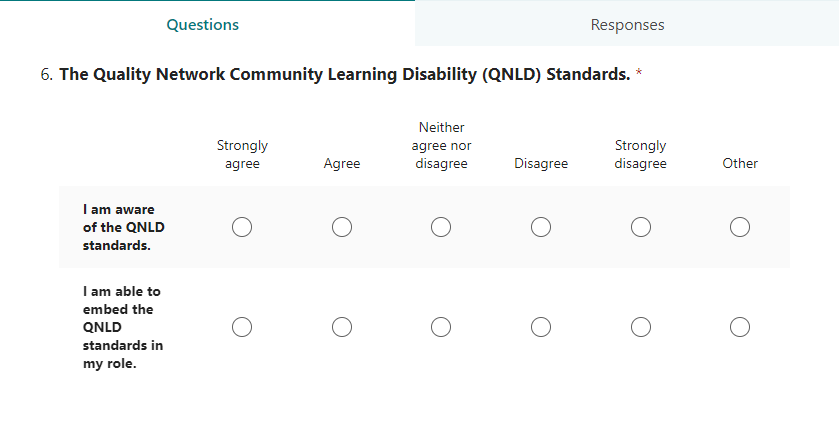
**

**
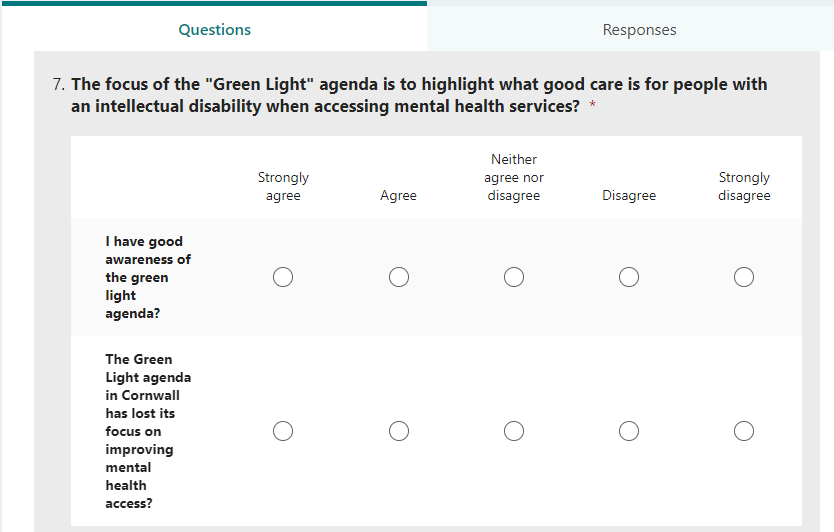
**

**
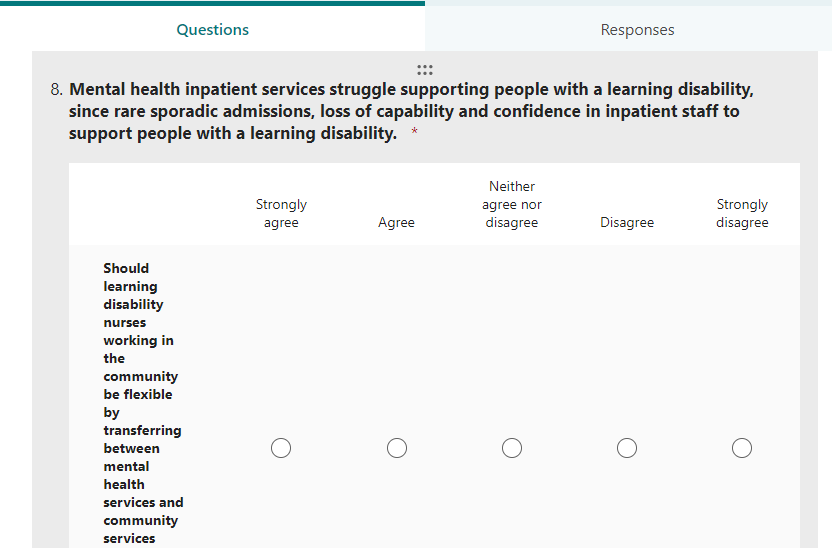
**

**
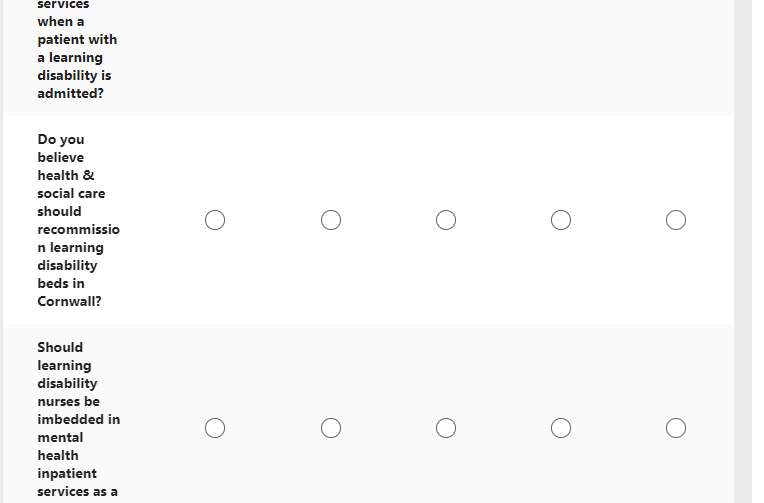
**

**
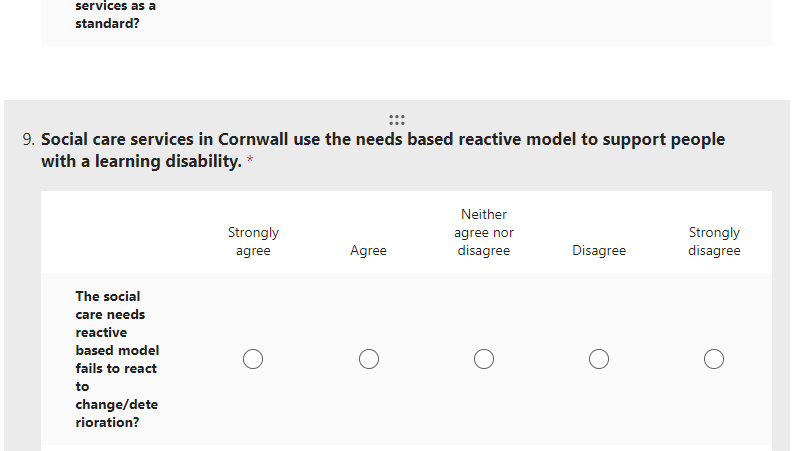
**

**
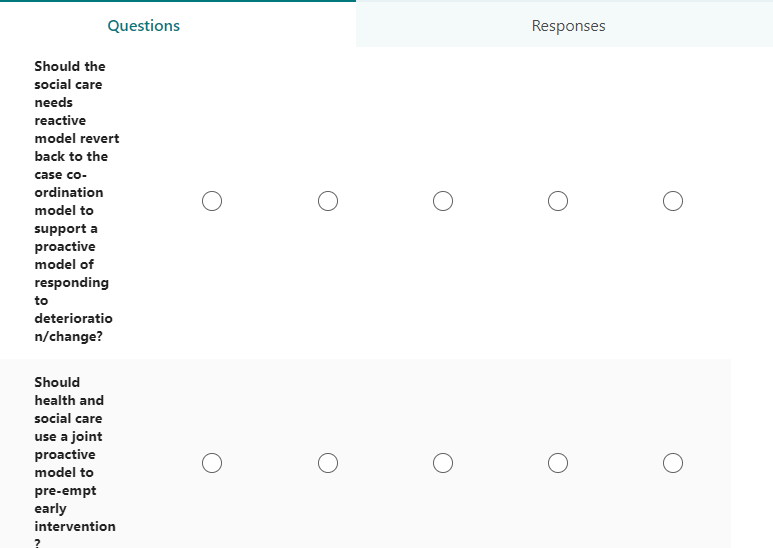
**

**
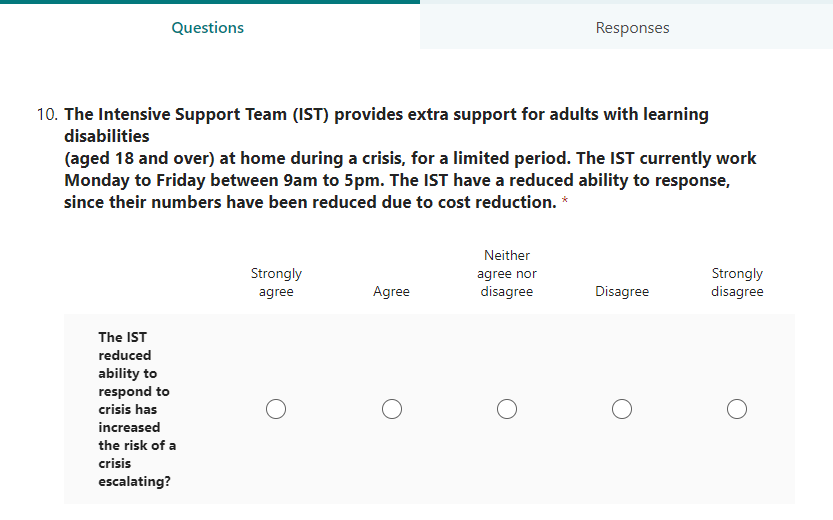
**

**
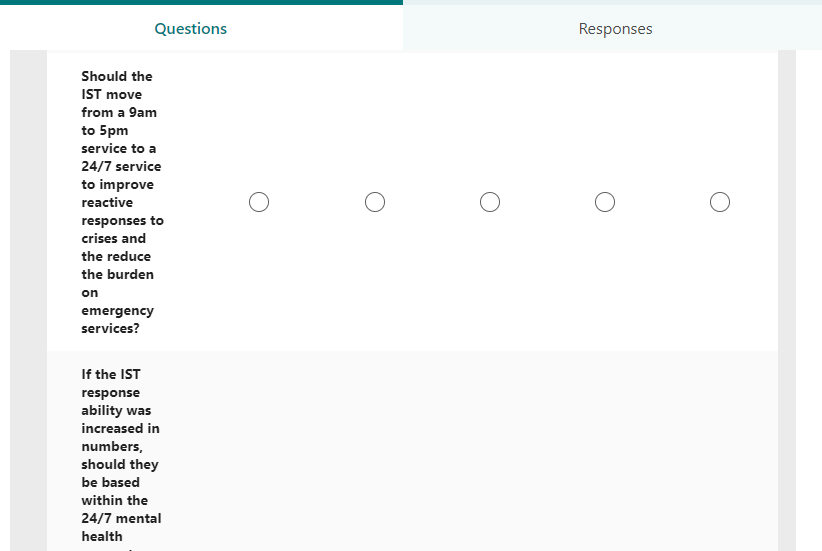
**

**
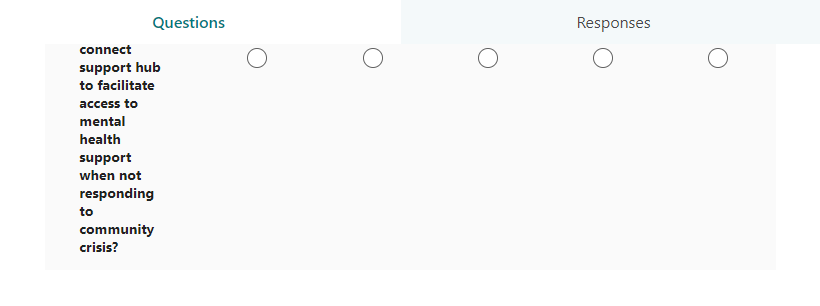
**

**
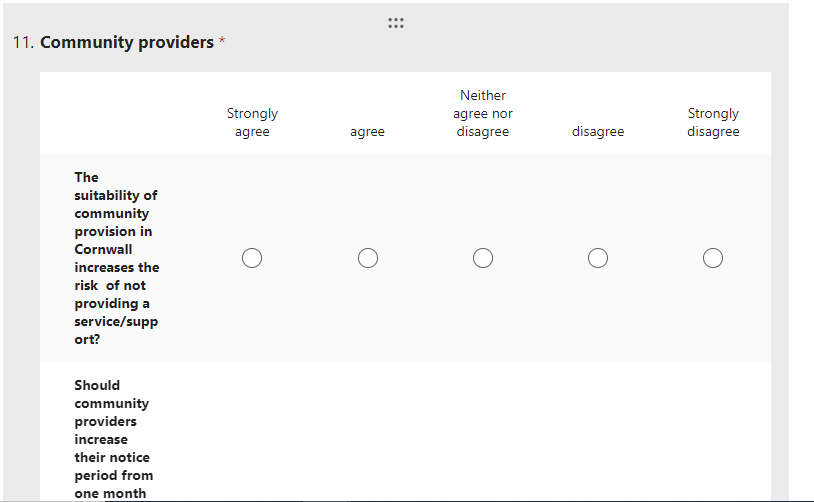
**

**
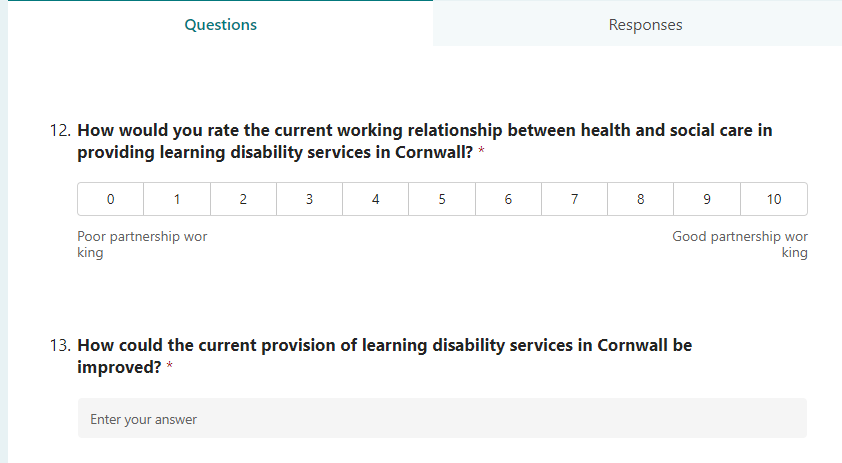
**

Supplement: sj-docx-5-isp-10.1177_00207640241303831 – Supplemental material for Understanding the current provisions of support for people with an intellectual disabilities and/or autism in crisis: A mixed methods study [file sj-docx-5-isp-10.1177_00207640241303831.docx]

## **Supplementary Material 6: Blue light & Deprived Areas of Cornwall**

**
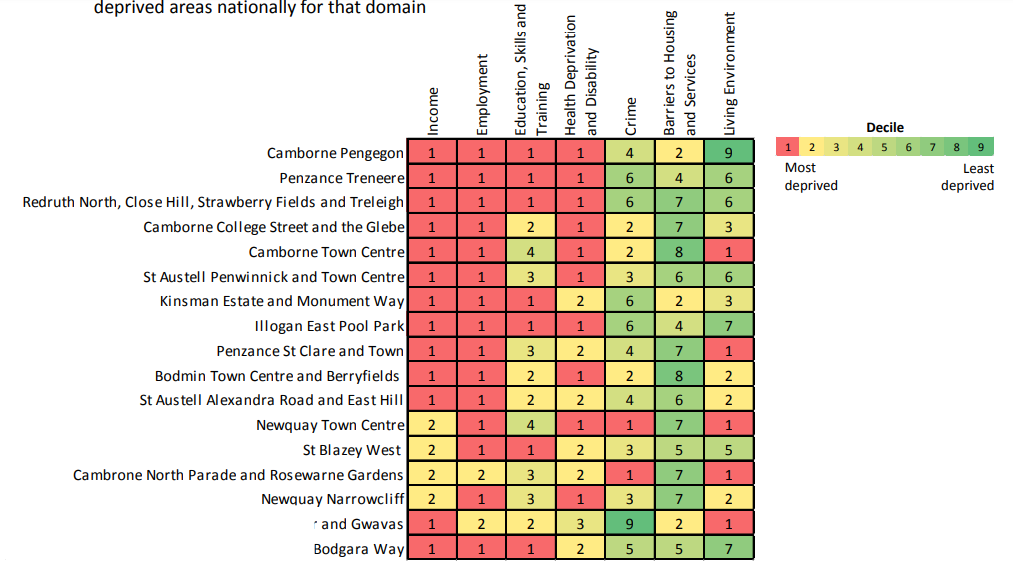
**

Supplement: sj-docx-6-isp-10.1177_00207640241303831 – Supplemental material for Understanding the current provisions of support for people with an intellectual disabilities and/or autism in crisis: A mixed methods study [file sj-docx-6-isp-10.1177_00207640241303831.docx]
