# Supplementary material for: Understanding the current provisions of support for people with an intellectual disabilities and/or autism in crisis: A mixed methods study
Source: Int J Soc Psychiatry. 2024 Dec 9;71(4):782–93. doi: 10.1177/00207640241303831 (PMC12171051; doi:10.1177/00207640241303831)
Supplement: sj-docx-3-isp-10.1177_00207640241303831 – Supplemental material for Understanding the current provisions of support for people with an intellectual disabilities and/or autism in crisis: A mixed methods study [file sj-docx-3-isp-10.1177_00207640241303831.docx]

## **Supplementary Material 3: Key Lines of Enquiry (KLOE) (adapted from:** [**https://www.england.nhs.uk/publication/care-and-treatment-review-key-lines-of-enquiry/**](https://www.england.nhs.uk/publication/care-and-treatment-review-key-lines-of-enquiry/)**)**

**
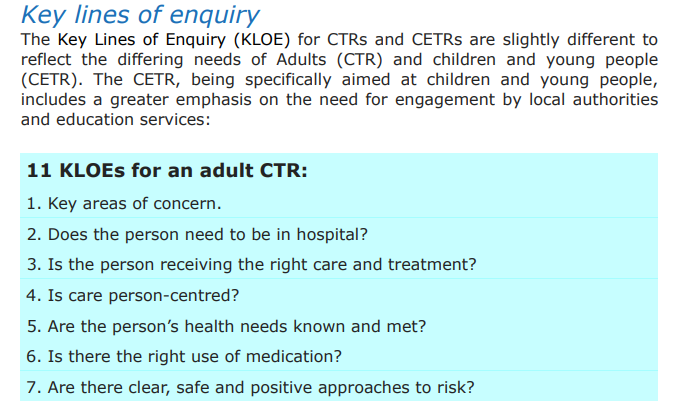
**

**
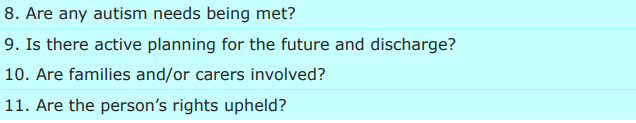
**
