# Supplementary material for: Understanding the current provisions of support for people with an intellectual disabilities and/or autism in crisis: A mixed methods study
Source: Int J Soc Psychiatry. 2024 Dec 9;71(4):782–93. doi: 10.1177/00207640241303831 (PMC12171051; doi:10.1177/00207640241303831)
Supplement: sj-docx-7-isp-10.1177_00207640241303831 – Supplemental material for Understanding the current provisions of support for people with an intellectual disabilities and/or autism in crisis: A mixed methods study [file sj-docx-7-isp-10.1177_00207640241303831.docx]

## **Supplementary Material 7:**

## **Key improvements suggestions by health and social care professionals**

- Longer notice period from community services, to allow the right support to be put in place.
- Proactive approach to peoples care to reduce crisis.
- Joint health and social care MDT’s
- Speciality social workers in learning difficulties needs to be recommissioned.
- Better investment and training for community health and social care employees including private care, to prevent admissions.
- Move away from excluding people from services.
- Better housing options for people with a learning difficulty.
- Health and social care teams working together as one service (Joint working).
- Having shared health and social care access to patient information i.e., access to NHS’s RIO and Cornwall Councils Mosaic and access to private care systems.
- Understanding each other’s professional roles and remits within these roles.
- Improved learning disability training in all services
- Improved access to community provision for learning disability services.
- Better and improved community provision, which a standard of provision is bench marked against PBS, Active support, Communication, and level of understanding learning disability.
- A move away from the medical model to a social care/community needs model.
- Investment in staff and IT.
- Greater working relationships with health and social care and consistency of teams.
- Improved pay for community workers, so retention is improved and consistency of workforce.
